# Effect of non-surgical periodontal therapy on concentrations of salivary biomarkers in patients with chronic periodontitis: A clinical trial

**DOI:** 10.15171/japid.2019.002

**Published:** 2019-08-31

**Authors:** Sahar Jabali, Mehran Mesgari Abbasi, Amir Ardalan Abdollahi

**Affiliations:** ^1^Department of Periodontics, Faculty of Dentistry, Urmia University of Medical Sciences, Urmia, Iran; ^2^Drug Applied Research Center, Tabriz University of Medical Science, Tabriz, Iran; ^3^Department of Endodontics, Faculty of Dentistry, Urmia University of Medical Sciences, Urmia, Iran

**Keywords:** Biomarkers, chronic periodontitis, dental scaling, root planing, saliva

## Abstract

**Background:**

Certain salivary biomarkers that are considered unique in relation to the physiological aspects of periodontitis can be helpful in the diagnosis of periodontitis by considering quantitative changes in such biomarkers. This study was undertaken to answer the question to what extent non-surgical periodontal treatment can affect concentrations of salivary biomarkers in patients suffering from chronic periodontitis.

**Methods:**

Eighteen patients with generalized moderate-to-severe chronic periodontitis were recruited for this study by considering periodontal parameters of gingival index (GI), probing pocket depths (PPD), clinical attachment levels (CAL) and a number of radiographic parameters. Salivary samples were analyzed at baseline and at one-month interval after non-surgical periodontal treatment consisting of scaling and root planing. Concentrations of salivary biomarkers, including cortisol, immunoglobulin A (Ig A), IL-6, interferon-γ, soluble intercellular adhesion molecule-1 (sICAM) and ALP, were determined with the use of an ELISA kit. Data were subjected to statistical analyses using paired t-test, with SPSS 15. Statistical significance was set at P<0.05.

**Results:**

Mean levels of IgA and interferon-γ decreased significantly after treatment (P<0.05); however, cortisol concentrations increased significantly after treatment. In addition, the decrease in IL-6, sICAM-1 and ALP levels were not significant (P>0.05).

**Conclusion:**

The results showed that the salivary levels of IgA and interferon-γ decreased and those of cortisol increased significantly subsequent to scaling and root planing.

## Introduction

A chronic inflammatory process induced by bacterial plaque accumulation in the gingival sulcus, leading to induction of an inflammatory response, is called periodontal disease.^1^ In addition to direct bacterial effect on periodontal tissues, indirect paths may also induce damage to the periodontium. Exposure of cells and underlying periodontal tissues occurs due to destruction of the periodontium by bacterial virulence factors. Therefore, bacterial constituents such as lipopolysaccharides (LPS) cause stimulation of the cells, including monocytes, lymphocytes and fibroblasts, leading to the release of some proinflammatory cytokines and mediators, including IL-6, IL-1, PGE-2, TNF-α and IL-8. The role of these cytokines is to induce inflammatory responses and catabolic processes alone or in coordination with each other.^2,3^ Soluble intercellular adhesion molecule-1 (sICAM) is a cell adhesion molecule which was identified and introduced by Seth et al in 1991 with the use of immunoblotting technique.^4^ The role of this cytokine is yet to be clarified; however, it is has been suggested that it has anti-inflammatory characteristics.^4,5^ ALP (alkaline phosphatase) is a homodimeric blood serum and salivary protein enzyme. Its activity has been reported to be at a maximum level in mixed saliva samples, with a minimum activity in parotid salivary samples.^6^ Cortisol, as one of the most important glucocorticoid, is produced in the adrenal cortex.^7^ Cortisol is found in the saliva because unbound serum cortisol enters the saliva through intracellular mechanisms and the major part of the salivary cortisol is unbound to protein.^8^ IgA is an antibody with an important role in the immune activity of mucous membranes. This IgA subclass is referred to as sIgA (secretory IgA) and is the predominant immunoglobulin in mucous secretions of salivary glands.^9^ IL-6 constitutes a family of cytokines, with a key role in the induction of the immune response to infections or traumas. Salivary IL-6 levels increase in periodontal diseases.^10^ Interferon-γ is a soluble dimer cytokine, also referred to as macrophage activating factor, has an important role in adaptive and innate immunity, especially against viral infections, intracellular bacteria and tumor control.^11,12^

Local host responses to microbial challenges are evaluated through cytokine levels.^13,14^ The whole saliva of patients with oral diseases is replete with various mediators that are responsible for chronic inflammation and tissue destruction. It is easy to collect salivary samples and no special equipment or trained operators are required; as a result, saliva is considered a valuable tool for the assessment of the concentration of its biomarkers.^15^ Furthermore, salivary biomarkers are more effective than those in the serum for certain diagnostic purposes.^16^ In addition, salivary biomarkers have been assessed to clarify the effect of smoking on periodontal health.^15^ Bertl et al studied the effect of non-surgical periodontal therapy on salivary melatonin levels and reported increases in these levels postoperatively, which correlated with decreases in the severity of periodontal inflammation.^17^ Furthermore, Onder et al reported that non-surgical periodontal treatment resulted in a decrease in oxidative stress biomarkers in patients suffering from chronic periodontitis.^18^

Scaling and root planing (SRP) are the gold standard of non-surgical periodontal treatment.^19^ The biomarkers selected in this study have been evaluated in several previous studies.^8,20,21^ Since no studies are available on the effect of SRP on the selected salivary biomarker concentrations, this study was undertaken to evaluate the impact of non-surgical periodontal therapy on concentrations of salivary biomarkers in subjects with chronic periodontitis.

## Methods

The protocol of this study was approved by the Ethics Committee of Tabriz University of Medical Sciences. A total of 18 subjects diagnosed with generalized moderate-to-severe chronic periodontitis, referring to the Department of Periodontics, Faculty of Dentistry, Tabriz University of Medical Sciences, from April 2014 to February 2015, were selected. The gap between the time of study and publishing the manuscript was due to the delay in gathering and analysis of the data. The following inclusion criteria were applied: patients with a minimum of 20 teeth in their oral cavities, gingival index of >1, a definitive diagnosis of generalized moderate-to-severe chronic periodontitis with at least 30% of the tooth-bearing areas having a minimum CAL of 3 mm and a PPD of ≥5 mm, with radiographic evidence of bone loss. The following exclusion criteria were applied: patients exhibiting systemic conditions, high blood pressure, aggressive periodontitis, pregnancy or breastfeeding, a history of taking systemic antibiotics in the past 6-month period, concurrent use of NSAIDs, a smoking habit, and a history of scaling and root planing in the previous 6-month period. The included subjects received adequate explanations about the mechanics of the study. Then the subjects submitted informed consent forms, followed by evaluation of GI, CAL and PPD parameters to confirm a diagnosis of periodontitis. The parameters were recorded at baseline and one month after intervention by a trained and calibrated operator (NA). A standard technique was used to determine PPD, i.e. the distance between the gingival sulcus and the deepest point that could be penetrated by a periodontal probe at mesiobuccal, mid-buccal, distobuccal and mid-lingual/mid-palatal areas, and recorded in a periodontal chart.

A periodontal probe was used in a standard technique to determine CAL from the CEJ to the most distal point of the sulcus or pocket penetrated by the probe.^22^

Gingival index (GI) is used to determine the extent and severity of gingivitis.^23^ Long-cone periapical radiographic technique was applied to measure bone loss to identify the subjects with chronic periodontitis.

### Collection of Salivary Samples

One week before salivary sampling, oral hygiene instructions were provided for the patients (modified Bass technique for brushing and use of dental floss). The patients were asked to be in a fasting state for 12 hours before submitting salivary samples after primary oral hygiene measures; 3 mL of salivary sample were collected from 8 a.m. to 10 a.m. SRP was performed using hand and ultrasonic instruments and if necessary, it was repeated in follow-up sessions for 4 weeks by a postgraduate student (S.J). O’Leary’s plaque index was recorded at baseline and postoperatively to achieve and maintain the index at <20% through instructing the subjects in hand plaque removal techniques.^24^ Salivary samples were also taken one-month interval after scaling and analyzed in the laboratory for salivary biomarkers. Unstimulated whole saliva samples were collected using passive drooling method; the participants used a straw to collect their salivary samples in a plastic vial. The samples underwent a centrifugation procedure at 3000 rpm for 10 minutes, followed by refrigeration at -70ºC until evaluations were carried out.

### Salivary Biomarker Assay

The concentrations of cortisol, Ig A, IL-6, interferon-γ, sICAM and ALP as salivary biomarkers were analyzed by double-antibody sandwich enzyme-linked immunosorbent assay (ELISA) kit (Glory Science Co., Ltd, USA). The assay unit was ng/mL for cortisol and sICAM, pg/mL for Ig B and interferon-γ, µg/mL for Ig A and u/L for alkaline phosphatase (ALP). The samples were analyzed in the Department of Immunology at Applied Drug Research Center, Tabriz University of Medical Sciences, Tabriz, Iran. The kit was conditioned at ambient temperature (18‒28°C) for 30 minutes prior to the assay.

### Statistical Analysis

Statistical analyses were implemented using SPSS 11.5. Kolmogorov–Smirnov test was used to assess normal distribution of data, which were expressed as means ± standard deviations (SD) for normally distributed data. Mean pre- and postoperative values were compared with paired t-test at P<0.05.

## Results

The mean age and BMI of the subjects in this study were 41.16±7.59 and 24.35±0.63, respectively. Of a total of 18 subjects in this study, 38.9% and 61.1% were male and female, respectively.

Table 1 presents the results of paired t-test in relation to comparison of salivary biomarker concentrations before and after treatment. The results showed that the levels of IgA and interferon-γ decreased significantly after treatment (P<0.05); however, cortisol concentrations increased significantly after treatment. In addition, the decreases in IL-6, sICAM-1 and ALP levels were not significant.

**Table 1:** The results of paired t-test for comparison of salivary biomarker concentrations before and after intervention

**Salivary biomarkers**	**No.**	**Mean ± SD**	**Degree of freedom**	**P-value**
		Before treatment After treatment		
**Cortisol**	18	5.83±2.56 11.10±2.30	17	<0.001*
**Ig A**	18	168.70±61.25 114.98±40.80	17	0.002*
**IL 6**	18	29.91±21.01 25.06±13.69	17	0.291
**Interferon-γ**	18	13.92±2041 3.05±9.08	17	0.041*
**sICAM-1**	14	15.16±16.44 10.39±6.14	13	0.349
**ALP**	18	21.38±8.04 19.66±6.55	17	0.493

*Statistically significant at P<0.05.

Furthermore, all the clinical parameters exhibited significant decreases after nonsurgical treatment (Table 2).

**Table 2:** The results of paired t-test for comparison of clinical variables before and after treatment

**Clinical variables**	**Mean ± SD**	**Degree of freedom**	**P-value**
	Before treatment After treatment	17	0.000*
**CAL (mm)**	5.0671±0.54 3.15±0.45	17	0.000*
**PPD (mm)**	4.67±0.51 2.63±0.45	17	0.000*
**GI**	1.85±0.27 0.71±0.33	17	0.000*
**PI (%)**	62.76±19.46 15.61±4.75	17	0.000*

*Statistically significant at P<0.05.

## Discussion

The present study evaluated the effect of non-surgical periodontal therapy on concentrations of salivary biomarker in patients diagnosed with chronic periodontitis. The results of this study revealed significant decreases in the levels of IgA and interferon-γ after treatment. Higher concentrations of IgA, Ig G and Ig M specific to periodontal pathogens have been shown in patients with periodontal disease compared with healthy patients. Also, the values decreased after periodontal treatment.^25^

Furthermore, the results of this showed that cortisol concentration significantly increased after treatment. Higher levels of salivary cortisol were recorded in patients suffering from severe periodontitis and emotional stress.^26^ According to previous studies, emotional stress is a periodontitis risk factor.^27^ Cortisol has important anti-inflammatory and immunosuppressive roles; it inhibits lymphocyte production and induces hyperplasia of lymphatic tissues. In addition, it inhibits production or antibodies.^7,28^ Anti-inflammatory properties of cortisol might be responsible for the increase in its salivary levels. Furthermore, the IL-6, sICAM-1 and ALP levels decreased after treatment but the decrease was not significant. Mole et al reported the highest values of sICAM−1 in gingival fluids in patients with adult periodontitis (AP) and the lowest in the group of healthy people.^21^ Nakamura and Slots reported the highest enzyme activities with ALP in the mixed whole saliva of patients with adult periodontitis compared to healthy individuals who exhibited the lowest enzyme activities.^29^ The higher salivary ALP in periodontitis could be attributed to alveolar bone loss, the main characteristics of periodontal disease. The low sample size of this study might be considered as a reason for non-significant results about the mentioned biomarkers. Moreover, similar to our previous study, the results of this study showed significant decreases in the clinical parameters of PI, GI, PPD and CAL after non-surgical intervention, indicating a decrease in the severity of inflammation.^30^ Saliva has biomarkers that are specific for physiologic aspects of periodontitis; as a result, quantitative changes in these markers might be considered as useful diagnostic tools.^31^ According to the results of this study, non-surgical periodontal treatment gave rise to significant decreases in some of the salivary biomarkers in patients with chronic periodontitis, which might be used as indicators for improvements in periodontal condition.

According to the limitations in the completion of the sample size and the time period of study, future studies should be carried out with larger sample sizes and in long-term prospective conditions in order to confirm the results of this study.

## Conclusions

Based on the results of this study, among the evaluated salivary biomarkers, decreases in the levels of IgA and interferon-γ and increases in cortisol levels were significant after non-surgical treatment in patients diagnosed with chronic periodontitis.
